# Rural-urban differences in workplace health promotion among employees of small and medium-sized enterprises in Germany

**DOI:** 10.1186/s12913-022-08052-9

**Published:** 2022-05-21

**Authors:** Lara Lindert, Lukas Kühn, Kyung-Eun Choi

**Affiliations:** 1 Center for Health Services Research, Brandenburg Medical School Theodor Fontane, Fehrbelliner Str. 38, 16816 Neuruppin, Germany; 2 Danube Private University (DPU) GmbH, Steiner Landstraße 124, 3500 Krems-Stein, Austria

**Keywords:** Occupational health, Psychosomatic complaints, Job satisfaction, Sick leave days, Employee health

## Abstract

**Background:**

Rural and urban areas hold different health challenges and resources for resident small and medium-sized enterprises (SMEs) and their employees. Additionally, residents of urban and rural areas differ in individual characteristics. This study aims at investigating potential rural-urban differences (1) in the participation rate in workplace health promotion (WHP) and (2) in the relationship of WHP and health relevant outcomes in residents living in rural or urban German areas and working in SMEs.

**Methods:**

Data of a large German Employee Survey in 2018 were used and analyzed by chi-square and t-tests and regression analyses regarding job satisfaction, sick days, and psychosomatic complaints. A total of 10,763 SME employees was included in analyses (23.9% living in rural, 76.1% living in urban areas).

**Results:**

Analyses revealed higher participation rates for SME employees living in rural areas. SME employees living in urban areas reported more often the existence of WHP. Results showed (a) significance of existence of WHP for psychosomatic complaints and (b) significance of participation in WHP for job satisfaction in SME employees living in urban but not for those living in rural areas.

**Conclusion:**

The revealed disparities of (1) higher participation rates in SME employees living in rural areas and in (2) the relationship of WHP aspects with health relevant outcomes are of special interest for practitioners (, e.g. human resource managers), politicians, and researchers by providing new indications for planning and evaluating WHP measures.

**Supplementary Information:**

The online version contains supplementary material available at 10.1186/s12913-022-08052-9.

## Introduction

Workplace health promotion (WHP) is a proven means in the health maintenance of employees. However, depending on the offer and personal characteristics, the participation rates vary enormously. To increase participation rates, WHP should be targeted to the needs of employees. Health and sickness absence data can help to identify relevant WHP topics in enterprises. Unfortunately, most smaller enterprises have limited access to such data (, e.g. from health insurances, human resource departments or occupational physicians) [1].

Demographic characteristics of target groups, workplace and work settings and extraneous context might be associated with WHP feasibility and sustainability [2]. Participation in WHP is influenced by social and environmental support, believe in effectiveness, time- and health-related barriers (, e.g. time of event during work or leisure time), fatiguing work and jobs with high physical or emotional demands with low job control [3–5].

Research on rural and urban aspects on WHP in small and medium-sized enterprises (SME) so far focuses on location of enterprises. In this study, we use a new approach and examine urban-rural differences in WHP based on employees residence for two reasons:

1. Rural and urban areas hold different challenges for both enterprises and residents.
2. All enterprises have access to information on employees’ place of residence.

Study results may especially help practitioners (, e.g. human resource managers,) in SME to identify preliminary indications for suitable WHP measures. WHP should be tailored to employees’ needs. An essential criterion for the suitability of WHP can be the place of employees’ residence. For example, employees living in the city with good access to training centers might rather make use of financial support for training courses, whereas employees living in rural areas, with no good access to training centers, might rather benefit from training possibilities at company sites. As WHP is of interest for SME worldwide [6–8] and as characteristics of urban and rural areas differ not only in Germany but worldwide [3, 9, 10], this study is of global interest. It approaches WHP aspects from a new perspective and provides orientation not only for practitioners, (e.g. human resource managers), but also for researchers in the field of work and health.

## Background

Demographic change and the shortage of skilled workers pose challenges for employers. It is becoming increasingly important for companies to keep employees healthy and on the job for as long as possible. Early detection of first symptoms as well as general prevention measures are enormously important to prevent chronic diseases. While the success of specific WHP measures is always context dependent, it is certain that WHP in general has positive effects. Studies revealed increased job satisfaction in connection with (offers of) WHP [6, 7, 11, 12] and positive effects of WHP measures on psychosomatic complaints [13]. Several studies revealed positive effects of WHP on sick leave and sickness costs [8, 11, 14, 15].

However, despite numerous studies on the positive effects of WHP, there is still a need to catch up: in particular, SMEs lag behind in the implementation of WHP [12, 16, 17]. To date, WHP has been predominantly found in large companies, although an upward trend can be seen in both areas. Interestingly, WHP offers are mainly used by employees in smaller companies: The direct employee approach is possibly easier in SMEs, and the small company size might facilitate motivation through colleagues. Social pressure might also “force” employees to participate in WHP measures. Moreover, WHP measures in large companies are probably more often offered only for selected departments and not open to all employees [12, 17]. Especially SMEs are affected by demographic change, as they often do not have resources to develop demographic management strategies and are rather located in rural areas [18].

Rural and urban areas hold different health challenges and resources for their residents: Urban areas can threaten residents’ health due to urban narrowness, lack of green spaces, high traffic volume with high noise levels, high air pollution, anonymity, and stress. On the other hand, they usually have a good local supply, a high density of public transport, good access to education, a rather health-promoting mobility, and a high density of health care facilities [19]. Companies in rural areas in particular are confronted with an aging workforce, as younger generations are increasingly drawn to urban areas [20, 21]. For rural areas, a systematic review revealed provider shortage, maldistribution, quality deficiencies, access limitations, and inefficient utilization as main aspects of health care shortage in developed countries. Accordingly, inefficient utilization is related to socio-cultural reasons: e.g. characteristics of rural residents like self-resilience, stoicism and proud [9, 22], and stigmatization of mental disorders [10] may hinder individuals in utilization of health care services in rural regions [23]. Furthermore, Young et al. [24] found that workers with bone fractures in rural areas are less likely to use care services and have shorter absences at work than workers in urban areas, which might be due to the accessibility of care services, but also due to psychosocial factors such as coping strategies and health attitudes: in this regard, the authors cited studies demonstrating that residents of rural areas have more active coping strategies, higher self-efficacy expectations, and accept adversity as part of rural life [25, 26].

## Hypotheses

Regarding the current state of research, our final research questions are: (1) *Are there differences in the use of WHP measures between employees of SME (SME-E) living in urban (SME-E*_*u*_) *and those living in rural (SME-E*_*r*_*) areas?* (2) *Are there differences in the relationship of existence of WHP with job satisfaction, psychosomatic complaints and sick days between SME-E*_*u*_ *and SME-E*_*r*_*?* (3) *Are there differences in the relationship of participation in WHP with job satisfaction, psychosomatic complaints and sick days between SME-E*_*u*_ *and SME-E*_*r*_*?*

Considering inefficient utilization of health care, characteristics of rural residents, and stigmatization aspects, it is assumed that.

*(h1) The use of WHP measures is more likely in SME-E*_*u*_*.*

According to regional differences in health-related environmental aspects and in residents’ characteristics, we hypothesize that.

*(h2) The relationship between existence of WHP with job satisfaction, psychosomatic complaints and sick days differs between SME-E*_*u*_ *and SME-E*_*r*_*.*

*(h3) The relationship between participation in WHP with job satisfaction, psychosomatic complaints and sick days differs between SME-E*_*u*_ *and SME-E*_*r*_*.*

## Methods

We used data of a cross-sectional employee survey that was conducted on behalf of the Federal Institute for Vocational Education and Training (BIBB) and the Federal Institute for Occupational Safety and Health (BAuA) from October 2017 to April 2018 via Computer Assisted Telephone Interviews (CATI) in Germany (BIBB/BAuA Employment Survey of the Working Population on Qualification and Working Conditions in Germany 2018, doi:10.7803/501.18.1.1.10). The data access was provided via a Scientific-Use-File [Remote Data Access; On-site Use in Bonn] of the Data Research Centre at the Federal Institute for Vocational Training and Education (BIBB-FDZ). The survey was aligned for German speaking paid employees, who were at least 15 years old and worked at least 10 hours per week. It covered topics on work requirements and activities, working conditions, health burdens and complaints, and qualifications. To recruit participants, a sampling frame was initially established by the BIK Institute using a random digit dialing procedure. As some individuals are reachable only via mobile phone, the recruitment process followed a dual-frame approach to capture mobile-only data to a sufficient extent. The gross samples were allocated separately, drawn separately, but processed together in the fieldwork. The dual-frame approach leads to bias-free samples without lump effects, that meet the requirements for random samples based on probability theory (probability sampling). Interviewers were trained beforehand. A total of 20,012 interviews have been conducted during survey period [27].

### Study population

**Fig. 1:**
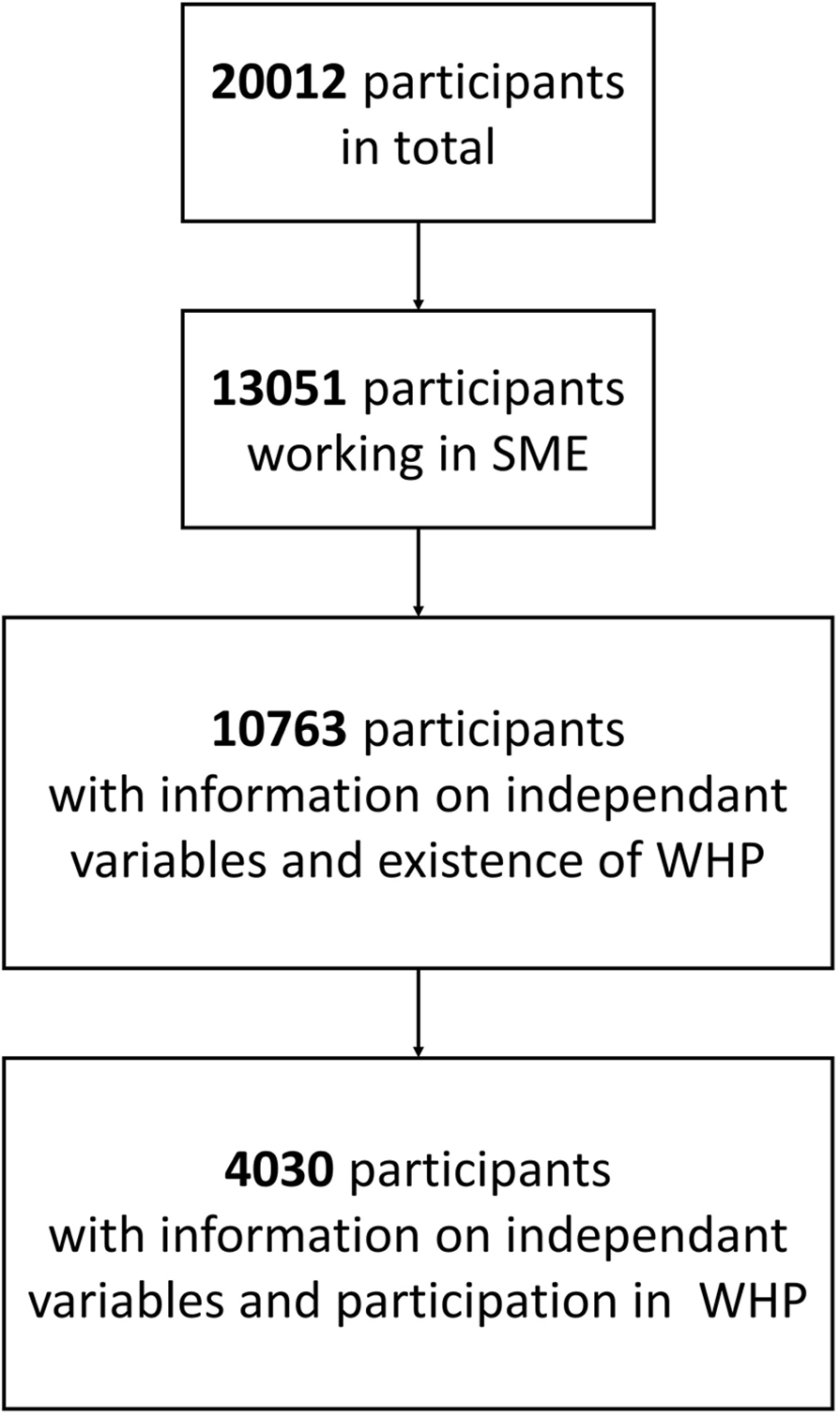
Flow chart of study sampling

In this study, we focused on data of participants with complete information on the following variables: SME, existence of WHP, job satisfaction, sick days, psychosomatic complaints, age, gender, educational status, career desire, private care tasks, emotional work, work intensity, leadership tasks, work life balance, and work duration. Companies with less than 250 employees (incl. trainees) where considered as SMEs. Data of employees in large companies (> 250 employees, incl. trainees) were excluded from this study (see Fig. 1).

### Measures

To distinguish SME-E in rural and urban areas, we created a dichotomous variable according to information on *BIK 10*. BIK 10 consists of 10 items and is labeled as follows: 1 = less than 2000 residents, 2 = 2000 to less than 5000 residents, 3 = 5000 to less than 20.000 residents, 4 = 20.000 to less than 50.000 residents, 5 = 50.000 to less than 100.000 residents (peripheral areas), 6 = 50.000 to less than 10.000 residents (core areas), 7 = 100.000 to less than 500.000 residents (peripheral areas), 8 = 100.000 to less than 500.000 residents (core areas), 9 = 500.000 and more residents (peripheral areas), 10 = 500.000 and more residents (core areas). Items 1 to 4 were considered as rural areas, items 5 to 10 as urban areas [28]. To measure WHP, participants were asked if WHP measures were carried out in their company within the last two years, and, if so, if they participated in the measure(s). Job satisfaction was measured using one item “*And now all things considered: How satisfied are you with your work overall?*” with answers from 1 “*not satisfied*” to 4 “*very satisfied*”. Sick days were recorded for the last 12 months (self-reported by participants). Psychosomatic complaints were measured asking for frequently occurring general fatigue, dullness or exhaustion, headaches, stomach or digestive problems, nervousness or irritability, nocturnal sleep disorders, despondency, physical exhaustion and emotional exhaustion within the last twelve months during work or on working days. Possible answers were yes/no. The possible range reached from 0 (no complaints) to 8 (complaints in all areas). For more details see also [29].

### Statistical analyses

As the main aim of this study is to examine differences in SME-E_r_ and SME-E_u_, data analyses followed a descriptive comparative approach.

To answer (h1) we conducted a chi-square test for participation in WHP of SME-E_r_ and SME-E_u_.

To answer (h2) and (h3) we conducted block-wise multiple linear regression analyses for psychosomatic complaints and job satisfaction (dependent continuous variables) for SME-E_r_ and SME-E_u_ each. For sick days (count variable) we conducted poisson regressions for SME-E_r_ and SME-E_u_ each. As independent variables we focused on existence of WHP (binary variable) and participation in WHP (binary variable). We considered *p* < .05 as level of significance for *p*-values in our analyses.

It is already well examined that working conditions and work organization can affect the health of employees [30–39]. Therefore, we integrated working conditions and factors of work organization (work intensity, emotional work, leadership tasks, work life balance, and work duration) as confounding variables in our regression models additionally to age, gender, education status, individuals’ career desire, and private care tasks. The results of block-wise analyses for psychosomatic complaints and job satisfaction can be found in supplementary files A and B. To test for multicollinearity, we examined correlations between variables. No value was found to be > .7 (see supplementary file C). According to the central limit theorem, the sampling distribution will be approximately normally distributed in large study samples [40–42].

Furthermore, we conducted chi-square and t-tests to reveal potential differences in existence of WHP, job satisfaction, psychosomatic complaints, sick days, and confounding variables between SME-E_u_ and SME-E_r_.

For this study, we decided to use the BIBB/BAuA Employment Survey 2018, since it

1. includes SME-E all over Germany,
2. provides information on participants residences according to BIK10,
3. provides information on existence of and participation in WHP, and delivers information on possibly confounding variables as
4. work relevant aspects as well as
5. individual aspects (, e.g. demographic data, private care tasks).

## Results

A total of 10,763 employees in SMEs with 2574 (23.9%) employees living in rural and 8189 (76.1%) living in urban areas remained. Mean age in rural areas was 47.7 (*SD* = 11.03) years, in urban areas 46.9 (*SD* = 11.44) years. In rural areas there were 1190 (46.2%) male and 1384 female (53.8%), in urban areas 3747 (45.8%) male and 4442 (54.2%) female employees. As participation in WHP could only be answered when existence of WHP was given, a total of 4030 participants remained for models including participation in WHP (see Table 1).

**Table 1:** Chi-square and t-tests regarding rural and urban residents

		N SME-E_r_ (percentage)				N SME-E_u_ (percentage)		chi-square test *p*-value
existence of WHP	yes / no	919 (35.7) / 1655 (64.3)				3122 (38.1) / 5067 (61.9)		**0.027**
participation in WHP	yes / no	648 (70.6) / 270 (29.4)				2025 (65.1) / 1087 (34.9)		**0.002**
gender	male / female	1190 (46.2) / 1384 (53.8)				3747 (45.8) / 4442 (54.2)		0.673
care	yes / no	231 (9.0) / 2343 (91.0)				637 (7.8) / 7552 (92.2)		0.052
leadership	yes / no	924 (35.9) / 1650 (64.1)				2811 (34.3) / 5378 (65.7)		0.144
		N	M	SD	Median (min / max)	95% confidence intervall		t-test *p*-value
						lower value	upper value	
job satisfaction	rural	2574	3.26	0.62	3.00 (1.00 / 4.00)	0.00194	0.05803	**0.036**
	urban	8189	3.23	0.64	3.00 (1.00 / 4.00)			
sick days	rural	2574	11.58	28.31	3.00 (0.00 / 365.00)	−0.12875	2.34173	0.079
	urban	8189	10.48	26.49	3.00 (0.00 / 365.00)			
psychosomatic complaints	rural	2574	2.40	2.37	2.00 (0.00 / 8.00)	−0.06421	0.14495	0.449
	urban	8189	2.36	2.36	2.00 (0.00 / 8.00)			
age	rural	2574	47.66	11.03	50.00 (16.00 / 78.00)	0.28529	1.27141	**0.002**
	urban	8189	46.88	11.44	49.00 (15.00 / 81.00)			
career desire	rural	2574	2.41	1.22	2.00 (1.00 / 5.00)	−0.11480	−0.00555	**0.031**
	urban	8189	2.47	1.26	2.00 (1.00 / 5.00)			
education	rural	2574	2.59	0.94	2.00 (1.00 / 4.00)	−0.25361	−0.16859	**0.000**
	urban	8189	2.80	1.03	2.00 (1.00 / 4.00)			
work intensity	rural	2574	3.09	0.62	3.20 (1.00 / 4.00)	−0.03335	0.02042	0.637
	urban	8189	3.09	0.60	3.20 (1.00 / 4.00)			
emotional work	rural	2574	2.40	0.99	3.00 (1.00 / 4.00)	−0.03433	0.05350	0.669
	urban	8189	2.39	0.99	2.00 (1.00 / 4.00)			
work life balance	rural	2574	3.49	0.73	4.00 (2.00 / 4.00)	−0.06220	0.00092	0.060
	urban	8189	3.52	0.71	4.00 (2.00 / 4.00)			
work duration	rural	2574	37.77	12.24	40.00 (10.00 / 120.00)	−0.43800	0.63800	0.715
	urban	8189	37.67	11.80	40.00 (10.00 / 120.00)			

*N* number of individuals in study population, *SME-E*_*r*_ employees of small and medium sized enterprises living in rural areas, *SME-E*_*u*_ employees of small and medium sized enterprises living in urban areas, *WHP* workplace health promotion, *M* mean, *SD* standard deviation; *p*-values < 0.05 are shown in bold

Job satisfaction is higher in SME-E_r_ (*p* < 0.05). In this sample there are no significant differences in gender, private care tasks, leadership function, psychosomatic complaints, sick days, work intensity, emotional work, work life balance, and work duration of SME-E_r_ and SME-E_u_. SME-E_r_ are higher in age (*p* < 0.01) and have lower career desire (*p* < 0.05) and education status (*p* < 0.001). SME-E_u_ reported more often to get WHP offers (*p* < 0.05). 35.7% of rural and 38.1% of urban residents reported existence of WHP within the last two years, of which 70.6% of rural and 65.1% of urban residents reported participation in WHP (see Table 1).

***(h1): The use of WHP measures is more likely in SME-E***_***u***_***.*** A chi-square test revealed that participation in WHP is more common in SME-E_r_ (*p* < 0.01) (see Table 1).

***(h2):*** ***The relationship between existence of WHP and job satisfaction, psychosomatic complaints, and sick days differs between SME-E***_***u***_ ***and SME-E***_***r***_**.** In multiple linear regression analyses for job satisfaction and psychosomatic complaints the existence of WHP in SMEs was significant for job satisfaction in SME-E_r_ (beta = 0.142, *p* < 0.001) and SME-E_u_ (beta = 0.132, *p* < 0.001) and for psychosomatic complaints in SME-E_u_ (beta = − 0.238, *p* < 0.001). No significance for existing WHP offers was found regarding psychosomatic complaints in SME-E_r_ (see Table 2). Existence of WHP for sick days was revealed in SME-E_u_ (95% CI, .887 to .912) but not in SME-E_r_ (95% CI, .987 to 1.036) (see Table 3).

**Table 2:** Multiple linear regression analyses, existence of WHP (N SME-E_r_ = 2574; N SME-E_u_ = 8189)

determinant factors	job satisfaction				psychosomatic complaints			
	beta (SE)		R^2^ (adjusted R^2)^		beta (SE)		R^2^ (adjusted R^2)^	
	SME-E_r_	SME-E_u_	SME-E_r_	SME-E_u_	SME-E_r_	SME-E_u_	SME-E_r_	SME-E_u_
existence of WHP	**0.142 (0.024)**	**0.132 (0.014)**	0.109 (0.105)	0.113 (0.111)	− 0.122 (0.084)	**− 0.238 (0.046)**	0.269 (0.266)	0.264 (0.263)
emotional work	**−0.092 (0.013)**	**− 0.087 (0.007)**			**0.842 (0.045)**	**0.755 (0.025)**		
work intensity	**−0.098 (0.021)**	**− 0.128 (0.013)**			**0.855 (0.074)**	**0.828 (0.042)**		
leadership tasks	**0.178 (0.026)**	**0.169 (0.015)**			**−0.321 (0.091)**	**−0.396 (0.05)**		
work life balance	**0.147 (0.017)**	**0.156 (0.01)**			**−0.393 (0.059)**	**−0.487 (0.033)**		
work duration	**0.004 (0.001)**	**0.003 (0.001)**			−0.001 (0.004)	**0.005 (0.002)**		
age	0.000 (0.001)	**0.003 (0.001)**			**−0.013 (0.004)**	**−0.017 (0.002)**		
gender	**0.107 (0.027)**	**0.081 (0.015)**			0.128 (0.092)	**0.304 (0.05)**		
education	**0.043 (0.013)**	**0.027 (0.007)**			**−0.182 (0.044)**	**−0.167 (0.022)**		
career desire	−0.015 (0.01)	**0.024 (0.006)**			−0.041 (0.036)	**−0.067 (0.02)**		
care	−0.024 (0.041)	0.001 (0.025)			0.2 (0.141)	**0.321 (0.084)**		

*SME-E*_*r*_ employees of small and medium sized enterprises living in rural areas, *SME-E*_*u*_ employees of small and medium sized enterprises living in urban areas, *WHP* workplace health promotion, *SE* standard error; *p*-values < 0.05 are shown in bold

**Table 3:** Poisson regression, existence of WHP (N SME-Er = 2574; N SME-Eu = 8189)

determinant factors	SME-E_r_			SME-E_u_		
	Exp(B)	95% Wald Confidence Interval		Exp(B)	95% Wald Confidence Interval	
		lower	upper		lower	upper
existence of WHP (no)	1.012	0.987	1.036	**0.900**	**0.887**	**0.912**
existence of WHP (yes)	1			1		
emotional work	**1.123**	**1.109**	**1.137**	**1.238**	**1.229**	**1.248**
work intensity	**1.361**	**1.331**	**1.391**	**1.115**	**1.101**	**1.129**
leadership tasks (no)	**1.584**	**1.541**	**1.627**	**1.284**	**1.265**	**1.304**
leadership tasks (yes)	1			1		
work life balance	**0.917**	**0.903**	**0.932**	**0.899**	**0.890**	**0.907**
work duration	**1.003**	**1.002**	**1.004**	**1.005**	**1.004**	**1.005**
age	**1.010**	**1.009**	**1.011**	**1.013**	**1.012**	**1.014**
male	**1.273**	**1.240**	**1.307**	**0.947**	**0.933**	**0.961**
female	1			1		
education	**0.828**	**0.817**	**0.839**	0.758	0.753	0.763
career desire	**0.928**	**0.918**	**0.937**	0.948	0.942	0.954
care (no)	**0.720**	**0.696**	**0.746**	0.926	0.905	0.948
Care (yes)	1			1		

Dependent variable: sick days

*SME-E*_*r*_ employees of small and medium sized enterprises living in rural areas, *SME-E*_*u*_ employees of small and medium sized enterprises living in urban areas, *WHP* workplace health promotion, *Exp(B)* exponentiated B; *p*-values < 0.05 are shown in bold

***(h3): The relationship between participation in WHP and job satisfaction, psychosomatic complaints, and sick days differs between SME-E***_***u***_ ***and SME-E***_***r***_***.*** Results of multiple linear regression analyses in only SME-E who reported existing WHP offers, showed significant results for participation in WHP on job satisfaction in SME-E_u_ (beta =0.07, *p* < 0.001) and not for psychosomatic complaints (see Table 4). Significance of participation in WHP was found for sick days in SME-E_r_ (95% CI, 1.110 to 1.206) and SME-E_u_ (95% CI, 1.063 to 1.112) (see Table 5).

**Table 4:** Multiple linear regression analyses, participation in WHP (N SME-E_r_ = 918; N SME-E_u_ = 3112)

determinant factors	job satisfaction				psychosomatic complaints			
	beta (SE)		R^2^ (adjusted R^2)^		beta (SE)		R^2^ (adjusted R^2)^	
	SME-E_r_	SME-E_u_	SME-E_r_	SME-E_u_	SME-E_r_	SME-E_u_	SME-E_r_	SME-E_u_
participation in WHP	0.066 (0.04)	**0.07 (0.022)**	0.091 (0.080)	0.087 (0.083)	−0.086 (0.148)	0.078 (0.075)	0.272 (0.264)	0.238 (0.235)
emotional work	**−0.077 (0.021)**	**− 0.088 (0.012)**			**0.833 (0.076)**	**0.729 (0.041)**		
work intensity	**−0.08 (0.034)**	**− 0.1 (0.02)**			**1.001 (0.127)**	**0.777 (0.069)**		
leadership tasks	**0.158 (0.04)**	**0.165 (0.023)**			−0.231 (0.149)	**−0.389 (0.079)**		
work life balance	**0.142 (0.028)**	**0.125 (0.016)**			**−0.476 (0.103)**	**−0.455 (0.056)**		
work duration	0.004 (0.002)	**0.003 (0.001)**			−0.01 (0.007)	**0.008 (0.004)**		
age	−0.002 (0.002)	**0.003 (0.001)**			− 0.007 (0.007)	**−0.012 (0.004)**		
gender	0.032 (0.042)	**0.067 (0.023)**			0.044 (0.153)	**0.228 (0.079)**		
education	0.027 (0.02)	0.014 (0.01)			**−0.198 (0.074)**	**−0.162 (0.036)**		
career desire	−0.015 (0.016)	**0.027 (0.009)**			−0.043 (0.06)	−0.056 (0.032)		
care	−0.088 (0.061)	0.025 (0.039)			0.05 (0.225)	**0.44 (0.134)**		

*SME-E*_*r*_ employees of small and medium sized enterprises living in rural areas, *SME-E*_*u*_ employees of small and medium sized enterprises living in urban areas, *WHP* workplace health promotion, *SE* standard error; *p*-values < 0.05 are shown in bold

**Table 5:** poisson regression, participation in WHP (N SME-Er = 918; N SME-Eu = 3112)

determinant factors	SME-E_r_			SME-E_u_		
	Exp(B)	95% Wald Confidence Interval		Exp(B)	95% Wald Confidence Interval	
		lower	upper		lower	upper
participation in WHP (no)	**1.157**	**1.110**	**1.206**	**1.087**	**1.063**	**1.112**
participation in WHP (yes)	1			1		
emotional work	**1.088**	**1.064**	**1.112**	**1.196**	**1.181**	**1.210**
work intensity	**1.231**	**1.185**	**1.279**	**1.054**	**1.033**	**1.077**
leadership tasks (no)	**1.501**	**1.436**	**1.570**	**1.313**	**1.281**	**1.346**
leadership tasks (yes)	1			1		
work life balance	0.989	0.960	1.018	**0.892**	**0.878**	**0.906**
work duration	**0.995**	**0.993**	**0.997**	0.999	0.998	1.000
age	**1.024**	**1.022**	**1.026**	**1.017**	**1.016**	**1.018**
male	**1.351**	**1.291**	**1.414**	**0.846**	**0.826**	**0.866**
female	1			1		
education	**0.903**	**0.883**	**0.922**	**0.755**	**0.746**	**0.763**
career desire	**1.049**	**1.031**	**1.067**	1.003	0.993	1.013
care (no)	**0.881**	**0.829**	**0.937**	**0.906**	**0.874**	**0.939**
care (yes)	1			1		

Dependent variable: sick days

*SME-E*_*r*_ employees of small and medium sized enterprises living in rural areas, *SME-E*_*u*_ employees of small and medium sized enterprises living in urban areas, *WHP* workplace health promotion, *Exp(B)* exponentiated B; *p*-values < 0.05 are shown in bold

Confounding variables were found to be significant in most cases, however beta and significance level differed (1) between models for job satisfaction and psychosomatic complaints and (2) between models for SME-E_r_ and SME-E_u_. The multiple linear regression model with all independent variables works best for psychosomatic complaints (*R*^*2*^ between 0.238 and 0.272) (see Tables 2 and 4). Models for sick days (see Tables 3 and 5) were all significant with *p* < .001.

## Discussion

Previous research identified positive effects of WHP on job satisfaction, psychosomatic complaints, and sick days [6–8, 11–15]. In this study, we found existence of and participation in WHP to be significant in some cases for psychosomatic complaints, job satisfaction, and sick days. This section will discuss how this, and differences in participation rates, might be explained by rural-urban differences in SME-E_r_ and SME-E_u_.

Despite our assumptions, SME-E_r_ might use WHP offers rather than SME-E_u_ to compensate for missing health promotion offers in rural areas. Furthermore, as even existing health services in rural areas are often not used [23], in our study, this seems to be different in relation to WHP. While health services are not used due to characteristics of rural residents and stigmatization of illness [9, 10, 22, 24], WHP might be associated with less stigmatization. However, future research needs to examine individual and environmental factors that affect participation in WHP with special focus on rural and urban aspects of employees’ residence.

With regard to significance of existence of WHP for psychosomatic complaints in SME-E_u_ but not in SME-E_r_, a possible explanation might be social network differences: SME-E_r_ might have a better social network, which is an important resource when it comes to psychological health [43]. However, no significant differences in psychosomatic complaints were found in study sample (see Table 1). Also, it might be the case that WHP offers reaching SME-E_u_ differ from offers reaching SME-E_r_ as they focus more on psychological aspects. It might also be, that SME-E_r_ do not use those offers due to stigmatization of mental disorders in rural regions [10]. However, no significance was found for participation in WHP neither in SME-E_r_ nor in SME-E_u_ for psychosomatic complaints. Sick days were found to be significantly lower in SME-E_u_ perceiving no existence of WHP, but not in SME-E_r_. This is against the results of studies revealing positive impact of WHP on sick days [8, 11, 14, 15]. However, this result is based on existence of, not on participation to, WHP. Employees with lower sick days might not be aware of WHP offers, as they perceive no need to actually work on their health. As Young et al. [24] found that workers with bone fractures in rural areas have shorter absences at work than workers in urban areas, this might also explain different results in SME-E_r_ and SME-E_u_ in this case.

Significant results for participation in WHP for job satisfaction in SME-E_u_ but not SME-E_r_ (b) might be explained by higher job satisfaction in SME-E_r_ compared to SME-E_u_ (see Table 1). As Fritz [13] reported significance of WHP for psychosomatic complaints, this is not confirmed by study results in case of WHP participation in both groups. However, this might be explained, as we have no information on quality, intention, and quantity of WHP offers, and as results of which Fritz [13] reported are based on a targeted intervention with 12 measures implemented.

Positive effects of WHP on sick leave and sickness costs have been revealed in past research [8, 11, 14, 15] and were confirmed by study results on participation in WHP for SME-E_u_ and SME-E_r_.

SMEs that offer WHP measures might rather have an overarching occupational health management and focus on employees’ health not only with WHP offers, but also when it comes to working conditions and requirements, which might explain differences in results for existence of WHP and participation in WHP regarding psychosomatic complaints and job satisfaction.

In line with previous research [30–39] confounding variables were mostly significant in all models for job satisfaction, psychosomatic complaints and sick days. Block-wise analyzes slightly showed changes in beta for existence of WHP and participation in WHP regarding job satisfaction and psychosomatic complaints. However, in most cases, significant results still remained significant – so existence of WHP and participation in WHP are partly but not totally mediated by confounding variables.

### Strengths and limitations

A major strengths of the study is the study population. By focusing on employees throughout Germany, the survey reached exactly the target group, that is relevant for research questions of this study. The methodological approach also ensured a good representativeness for German employees.

However, to participate in the interviews, individuals had to be German speaking. This may have biased the results as non-German-speaking persons were not represented in study sample. The data do also not provide information on intention, quality and quantity of WHP measures and participation. Another limitation emerges from the subjective perspective of employees – WHP measures might have been offered in companies under different designations (, e.g. as occupational health and safety measure,) or without any clear label at all. Therefore, employees might have not reported the WHP measures in the interviews, since employees were not aware of having taken part in WHP offers [17]. Also differences in age, educational status, job satisfaction, and career desire have to be considered when focusing on different results in SME-E_u_ and SME-E_r_.

Despite the mentioned limitations, due to the quality of sampling process, study results can be transferred to German speaking SME-E_r_ and SME-E_u_ in Germany. In global context, study results give first hints for practitioners, e.g. human resource managers. When planning WHP measures, it might be useful not to focus on company location only, but also on employees’ residence. For example, SME-E_r_ might use training possibilities at company sites rather than SME-E_u_ as access to training centers in rural areas are limited. In this case, the decision whether to offer training opportunities for employees should depend on where most employees live and not on where the company is located. However, future research needs to clarify which aspects of urban or rural life exactly have an impact on employees’ participation rates and focus on content, quality, and quantity of WHP measures. Thereby, both – individual and environmental – factors should be considered.

As interviews were conducted prior to the outbreak of SARS-CoV-2, further research on WHP in rural and urban settings should also take into account the special challenges for employees during and after the pandemic.

## Conclusion

Research on rural and urban aspects on WHP in SME so far focuses on location of enterprises. In this study, we used a new approach and examined urban-rural differences in WHP, based on employees residence. Results indicate, that the place of residence influences the participation in WHP. When planning WHP measures, it might be useful not to focus on the company location only, but also on employees’ residence. Future research could examine specific needs of both rural and urban residents and how the currently prevailing supply meets these needs. Practitioners, politicians, as well as researchers are called upon to use these insights for the development of human resource management.

## Supplementary Information

**Additional file 1.**

## Data Availability

Data is free for scientific purpose and can be requested as scientific-use-file at the Federal Institute for Vocational Education and Training (https://www.bibb.de/de/1403.php, doi:10.7803/501.18.1.1.10).

## Abbreviations

BAUA: Federal Institute for Occupational Safety and Health
BIBB: Federal Institute for Vocational Education and Training
BIBB-FDZ: Data Research Centre at the Federal Institute for Vocational Training and Education
CATI: Computer Assisted Telephone Interviews
SMEs: Small and medium-sized enterprises
SME-E: Employees of small and medium-sized enterprises
SME-E_r_: Employees of small and medium-sized enterprises living in rural areas
SME-E_u_: Employees of small and medium-sized enterprises living in urban areas
WHP: Workplace health promotion
