# Supplementary material for: Rural-urban differences in workplace health promotion among employees of small and medium-sized enterprises in Germany
Source: BMC Health Serv Res. 2022 May 21;22:681. doi: 10.1186/s12913-022-08052-9 (PMC9123665; doi:10.1186/s12913-022-08052-9)
Supplement: Supplementary file 1 — Additional file 1. [file 12913_2022_8052_MOESM1_ESM.docx]

# Supplementary file A: block-wise regression analyses (existence of WHP)

Table A1: block-wise regression analyses, dependent variable: job satisfaction (N SME-E_r_ = 2574; N SME-E_u_ = 8189)

| determinant  factors | multiple linear regression analysis | | | | R^2^ (adjusted R^2)^ | |
| --- | --- | --- | --- | --- | --- | --- |
|  | beta (SE) | | p value | |  |  |
|  | SME-E_r_ | SME-E_u_ | SME-E_r_ | SME-E_u_ | SME-E_r_ | SME-E_u_ |
| Block I | | | | | | |
| existence of WHP | 0.161 (0.025) | 0.15 (0.014) | **0.000***** | **0.000***** | 0.015 (0.015) | 0.013 (0.013) |
| Block II | | | | | | |
| existence of WHP | 0.170 (0.025) | 0.158 (0.014) | **0.000***** | **0.000***** | 0.051 (0.050) | 0.057 (0.057) |
| emotional work | -0.090 (0.013) | -0.094 (0.007) | **0.000***** | **0.000***** |  |  |
| work intensity | -0.078 (0.021) | -0.113 (0.012) | **0.000***** | **0.000***** |  |  |
| Block III | | | | | | |
| existence of WHP | 0.139 (0.024) | 0.132 (0.014) | **0.000***** | **0.000***** | 0.098 (0.096) | 0.103 (0.102) |
| emotional work | -0.076 (0.013) | -0.076 (0.007) | **0.000***** | **0.000***** |  |  |
| work intensity | -0.091 (0.021) | -0.12 (0.012) | **0.000***** | **0.000***** |  |  |
| leadership tasks | 0.177 (0.026) | 0.182 (0.015) | **0.000***** | **0.000***** |  |  |
| work life balance | 0.154 (0.017) | 0.158 (0.01) | **0.000***** | **0.000***** |  |  |
| work duration | 0.002 (0.001) | 0.002 (0.001) | 0.072 | **0.001**** |  |  |
| Block IV | | | | | | |
| *see table 1* | | | | | | |
| SME-E_r_ = employees of small and medium sized enterprises living in rural areas; SME-E_u_ = employees of small and medium sized enterprises living in urban areas; WHP = workplace health promotion; SE = standard error; * p < 0.05; ** p < 0.01; *** p < 0.001. | | | | | | |

Table A2: block-wise regression analyses, dependent variable: psychosomatic complaints (N SME-E_r_ = 2574; N SME-E_u_ = 8189)

| determinant  factors | multiple linear regression analysis | | | | R^2^ (adjusted R^2)^ | |
| --- | --- | --- | --- | --- | --- | --- |
|  | beta (SE) | | p value | |  |  |
|  | SME-E_r_ | SME-E_u_ | SME-E_r_ | SME-E_u_ | SME-E_r_ | SME-E_u_ |
| Block I | | | | | | |
| existence of WHP | -0.127 (0.097) | -0.263 (0.054) | 0.191 | **0.000***** | 0.001 (0.000) | 0.003 (0.003) |
| Block II | | | | | | |
| existence of WHP | -0.217 (0.085) | -0.328 (0.047) | **0.011*** | **0.000***** | 0.240 (0.239) | 0.219 (0.218) |
| emotional work | 0.852 (0.045) | 0.801 (0.025) | **0.000***** | **0.000***** |  |  |
| work intensity | 0.842 (0.071) | 0.862 (0.041) | **0.000***** | **0.000***** |  |  |
| Block III | | | | | | |
| existence of WHP | -0.142 (0.085) | -0.253 (0.047) | 0.094 | **0.000***** | 0.259 (0.258) | 0.248 (0.248) |
| emotional work | 0.818 (0.044) | 0.746 (0.025) | **0.000***** | **0.000***** |  |  |
| work intensity | 0.856 (0.074) | 0.847 (0.042) | **0.000***** | **0.000***** |  |  |
| leadership tasks | -0.39 (0.09) | -0.478 (0.05) | **0.000***** | **0.000***** |  |  |
| work life balance | -0.407 (0.059) | -0.51 (0.034) | **0.000***** | **0.000***** |  |  |
| work duration | -0.004 (0.004) | -0.003 (0.002) | 0.263 | 0.172 |  |  |
| Block IV | | | | | | |
| *see table 1* | | | | | | |
| SME-E_r_ = employees of small and medium sized enterprises living in rural areas; SME-E_u_ = employees of small and medium sized enterprises living in urban areas; WHP = workplace health promotion; SE = standard error; * p < 0.05; ** p < 0.01; *** p < 0.001. | | | | | | |

# Supplementary file B: block-wise regression analyses (participation in WHP)

Table B1: block-wise regression analyses, dependent variable: job satisfaction (N SME-E_r_ = 918; N SME-E_u_ = 3112)

| determinant  factors | multiple linear regression analysis | | | | R^2^ (adjusted R^2)^ | |
| --- | --- | --- | --- | --- | --- | --- |
|  | beta (SE) | | p value | |  |  |
|  | SME-E_r_ | SME-E_u_ | SME-E_r_ | SME-E_u_ | SME-E_r_ | SME-E_u_ |
| Block I | | | | | | |
| participation in WHP | 0.074 (0.041) | 0.078 (0.022) | 0.073 | **0.001**** | 0.003 (0.002) | 0.004 (0.004) |
| Block II | | | | | | |
| participation in WHP | 0.081 (0.041) | 0.083 (0.022) | **0.048*** | **0.000***** | 0.038 (0.035) | 0.040 (0.039) |
| emotional work | -0.094 (0.02) | -0.094 (0.012) | **0.000***** | **0.000***** |  |  |
| work intensity | -0.056 (0.033) | -0.074 (0.019) | 0.095 | **0.000***** |  |  |
| Block III | | | | | | |
| participation in WHP | 0.062 (0.04) | 0.073 (0.022) | 0.122 | **0.001**** | 0.084 (0.078) | 0.079 (0.077) |
| emotional work | -0.075 (0.02) | -0.08 (0.011) | **0.000***** | **0.000***** |  |  |
| work intensity | -0.076 (0.034) | -0.091 (0.02) | **0.026*** | **0.000***** |  |  |
| leadership tasks | 0.151 (0.04) | 0.179 (0.022) | **0.000***** | **0.000***** |  |  |
| work life balance | 0.144 (0.028) | 0.124 (0.016) | **0.000***** | **0.000***** |  |  |
| work duration | 0.003 (0.002) | 0.003 (0.001) | 0.068 | **0.010*** |  |  |
| Block IV | | | | | | |
| *see table 1* | | | | | | |
| SME-E_r_ = employees of small and medium sized enterprises living in rural areas; SME-E_u_ = employees of small and medium sized enterprises living in urban areas; WHP = workplace health promotion; SE = standard error; * p < 0.05; ** p < 0.01; *** p < 0.001. | | | | | | |

Table B2: block-wise regression analyses, dependent variable: psychosomatic complaints (N SME-E_r_ = 918; N SME-E_u_ = 3112)

| determinant  factors | multiple linear regression analysis | | | | R^2^ (adjusted R^2)^ | |
| --- | --- | --- | --- | --- | --- | --- |
|  | beta (SE) | | p value | |  |  |
|  | SME-E_r_ | SME-E_u_ | SME-E_r_ | SME-E_u_ | SME-E_r_ | SME-E_u_ |
| Block I | | | | | | |
| participation in WHP | -0.032 (0.17) | 0.136 (0.085) | 0.851 | 0.111 | 0.000  (-0.001) | 0.001 (0.000) |
| Block II | | | | | | |
| participation in WHP | -0.101 (0.149) | 0.082 (0.076) | 0.496 | 0.282 | 0.241 (0.238) | 0.198 (0.198) |
| emotional work | 0.857 (0.074) | 0.779 (0.04) | **0.000***** | **0.000***** |  |  |
| work intensity | 0.977 (0.122) | 0.805 (0.067) | **0.000***** | **0.000***** |  |  |
| Block III | | | | | | |
| participation in WHP | -0.054 (0.147) | 0.107 (0.075) | 0.712 | 0.155 | 0.265 (0.260) | 0.225 (0.223) |
| emotional work | 0.802 (0.073) | 0.73 (0.04) | **0.000***** | **0.000***** |  |  |
| work intensity | 1.005 (0.126) | 0.78 (0.069) | **0.000***** | **0.000***** |  |  |
| leadership tasks | -0.282 (0.147) | -0.458 (0.078) | 0.056 | **0.000***** |  |  |
| work life balance | -0.488 (0.103) | -0.476 (0.056) | **0.000***** | **0.000***** |  |  |
| work duration | -0.013 (0.006) | 0.001 (0.004) | **0.042*** | 0.776 |  |  |
| Block IV | | | | | | |
| *see table 1* | | | | | | |
| SME-E_r_ = employees of small and medium sized enterprises living in rural areas; SME-E_u_ = employees of small and medium sized enterprises living in urban areas; WHP = workplace health promotion; SE = standard error; * p < 0.05; ** p < 0.01; *** p < 0.001. | | | | | | |

# Supplementary file C: correlations between variables

Table C1: correlations between variables, (N = 10763)

|  |  | 1 | 2 | 3 | 4 | 5 | 6 | 7 |
| --- | --- | --- | --- | --- | --- | --- | --- | --- |
| Work duration | Pearson-correlation |  | **-0.035***** | **0.204***** | **0.125***** | **0.241***** | **0.068***** | **-0.232***** |
|  | sig. (2-sided) |  | 0.000 | 0.000 | 0.000 | 0.000 | 0.000 | 0.000 |
| Age | Pearson-correlation | **-0.035***** |  | **-0.385***** | **0.079***** | -0.013 | **0.091***** | -0.002 |
|  | sig. (2-sided) | 0.000 |  | 0.000 | 0.000 | 0.192 | 0.000 | 0.854 |
| Career desire | Pearson-correlation | **0.204***** | **-0.385***** |  | **0.054***** | **0.107***** | **-0.028***** | **-0.049***** |
|  | sig. (2-sided) | 0.000 | 0.000 |  | 0.000 | 0.000 | 0.003 | 0.000 |
| Education | Pearson-correlation | **0.125***** | **0.079***** | **0.054***** |  | **0.121***** | **0.139***** | 0.012 |
|  | sig. (2-sided) | 0.000 | 0.000 | 0.000 |  | 0.000 | 0.000 | 0.225 |
| Work intensity | Pearson-correlation | **0.241***** | -0.013 | **0.107***** | **0.121***** |  | **0.374***** | **-0.220***** |
|  | sig. (2-sided) | 0.000 | 0.192 | 0.000 | 0.000 |  | 0.000 | 0.000 |
| Emotional work | Pearson-correlation | **0.068***** | **0.091***** | **-0.028***** | **0.139***** | **0.374***** |  | **-0.184***** |
|  | sig. (2-sided) | 0.000 | 0.000 | 0.003 | 0.000 | 0.000 |  | 0.000 |
| Work life balance | Pearson-correlation | **-0.232***** | -0.002 | **-0.049***** | 0.012 | **-0.220***** | **-0.184***** |  |
|  | sig. (2-sided) | 0.000 | 0.854 | 0.000 | 0.225 | 0.000 | 0.000 |  |

* p < 0.05; ** p < 0.01; *** p < 0.001.
